# Angiography-Guided Limb Salvage Planning in a Surgical Candidate With Gas Gangrene of the Foot

**DOI:** 10.7759/cureus.92935

**Published:** 2025-09-22

**Authors:** Murali K Manikkavelu, Venkatesh Aadhe, Raven Sands, Frederick Tiesenga, Mahamed Jama

**Affiliations:** 1 General Surgery, Community First Medical Center, Chicago, USA; 2 Medicine, Windsor University School of Medicine, Basseterre, KNA; 3 Medicine, All Saints Medical School, Roseau, DMA

**Keywords:** below the knee amputation, clostridial myonecrosis, ct angiogram, debridement, diabetic foot infection, gas gangrene, limb salvage, patient autonomy, surgical decision-making, vascular imaging

## Abstract

Gas gangrene is a fulminant soft-tissue infection that requires rapid intervention to prevent extensive tissue destruction and mortality. Diabetic patients with peripheral neuropathy often have delayed recognition of injuries and impaired wound healing, placing them at substantially higher risk of severe complications. This report presents a 60-year-old male patient with diabetes and peripheral neuropathy who developed gas gangrene of the right foot. Despite two initial debridements, concern remained for progressive infection necessitating limb amputation. An abdominal aortogram with run-off demonstrated preserved distal perfusion of the right lower extremity, allowing the surgical team to consider below-the-knee amputation (BKA) rather than above-the-knee amputation (AKA). BKA allows for improved prosthetic fitting and lower energy expenditure during ambulation, which is preferable to the outcomes typically seen with AKA. Ultimately, the patient chose to defer amputation and arranged for outpatient follow-up to revisit the decision at a later time. This case underscores the role of angiography in guiding amputation planning and highlights the need to balance clinical judgment with patient autonomy in limb salvage decisions.

## Introduction

Gas gangrene is a life-threatening necrotizing infection predominantly caused by *Clostridium perfringens*. Patients with diabetes mellitus are at increased risk due to their immunocompromised state, peripheral neuropathy, and vascular insufficiency, all of which delay wound recognition and impair healing [1,2]. Without immediate treatment, the condition is frequently fatal [1]. Initial management often begins with aggressive medical therapy, but surgical intervention is universally required to halt its rapid progression [3].

When amputation is required, careful preoperative planning is critical, as the selected level of amputation has profound implications for the patient’s quality of life. Preserving the knee joint with a below-the-knee amputation (BKA) offers major advantages, including improved prosthetic function, more efficient ambulation, and a reduced risk of complications such as contractures, skin breakdown, and muscle atrophy [4,5]. However, successful BKA depends on adequate distal perfusion to ensure proper wound healing.

In this context, angiography provides a pivotal role. By directly visualizing arterial flow and distal runoff, angiography allows the surgical team to make an evidence-based decision regarding the most appropriate amputation level [6]. This is particularly relevant in diabetic patients with gas gangrene, where the urgency of infection control must be balanced with the long-term benefits of limb preservation. In the case presented here, angiographic evaluation revealed preserved distal perfusion, supporting a BKA as a safe and feasible option. Without this imaging-guided planning, the patient would likely have undergone a more proximal above-the-knee amputation (AKA), with significant functional consequences [6].

## Case presentation

A 60-year-old man with a history of diabetes complicated by peripheral neuropathy and hypertension presented to the emergency department with a rapidly worsening wound on his right foot. He was previously ambulatory and reported a sedentary lifestyle, with no recent history of trauma or injury to the affected limb. The wound began a few days earlier but quickly became painful, foul-smelling, and darker in appearance. He also reported stopping his diabetes medications over the preceding days because he had run out.

On arrival, he appeared uncomfortable. He was afebrile at 97.8°F, with a heart rate of 68 bpm, blood pressure of 99/58 mmHg, respiratory rate of 20 breaths per minute, and oxygen saturation of 97% on room air. Examination revealed ulceration with purulent discharge on the dorsum of the right foot, discoloration and loss of sensation of the second and third toes, and surrounding erythema extending proximally with associated crepitus. Peripheral vascular exam showed palpable bilateral femoral and popliteal pulses, but dorsalis pedis and posterior tibial pulses on the right were diminished compared to the contralateral side. Capillary refill of uninvolved toes was delayed. The ankle-brachial index was not measured at presentation. No trophic skin changes such as hair loss or atrophy were observed on the contralateral foot, suggesting absence of chronic ischemia.

Admission laboratory studies are summarized in Table 1. Results were notable for leukocytosis, anemia, renal dysfunction, hyponatremia, hyperglycemia, elevated inflammatory markers, and elevated lactate, consistent with severe soft tissue infection and systemic involvement.

**Table 1:** Admission laboratory results BUN: Blood urea nitrogen; CRP: C-reactive protein; aPTT: Activated partial thromboplastin time; PT: Prothrombin time; INR: International normalized ratio

Test	Result	Reference Range
White Blood Cells (×10³/µL)	19.1	4.0-11.0
Hemoglobin (g/dL)	10.8	13.5-17.5
Hematocrit (%)	33.1	40-50
Platelets (×10³/µL)	505	150-450
Sodium (mmol/L)	130	135-145
Glucose (mg/dL)	176	70-110
Creatinine (mg/dL)	1.78	0.6-1.3
BUN (mg/dL)	34	7-20
Lactate (mmol/L)	3.1	0.5-2.0
CRP (mg/L)	287	<10
aPTT (sec)	30	25-35
PT (sec)	14.2	11-13.5
INR	1.2	0.8-1.2

**Figure 1:**
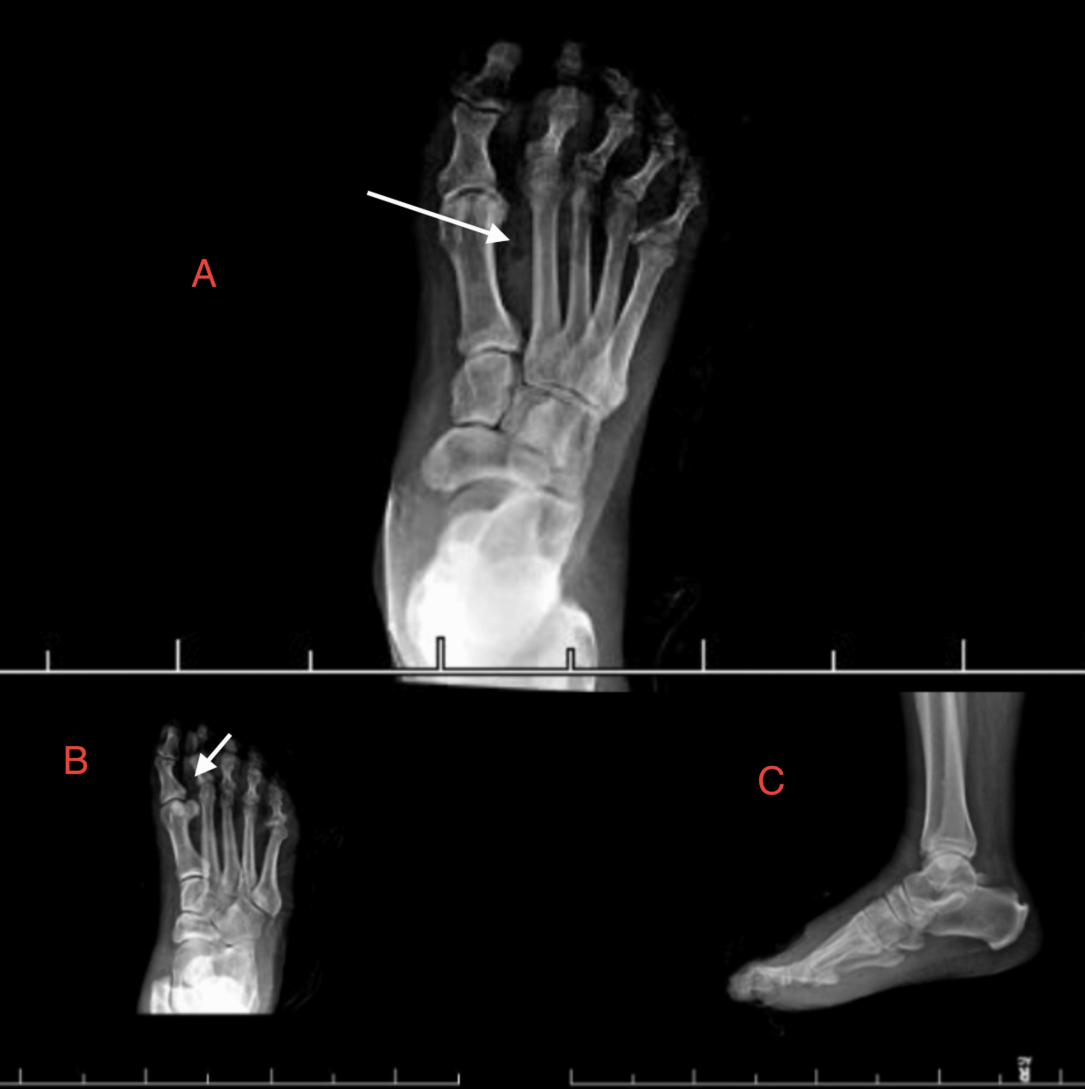
Initial right foot radiographs (A) Anteroposterior and (B) oblique views showing subcutaneous emphysema in the soft tissues (arrows), concerning for gas gangrene. Lateral view (C) provided for completeness, where the emphysema is less conspicuous. No definite radiographic evidence of osteomyelitis is seen.

Initial foot X-ray demonstrated subcutaneous emphysema concerning for gas gangrene, without definite evidence of osteomyelitis (Figure 1). The patient was started on intravenous vancomycin, piperacillin-tazobactam, and clindamycin. Later that day, he underwent surgical debridement with amputation of the right third toe. Necrotic tissue extended proximally along subcutaneous tissue and tendon into the plantar space. Penrose drains were placed and both wound cultures and a bone biopsy were obtained. The wound measured approximately 15×8 cm².

**Figure 2:**
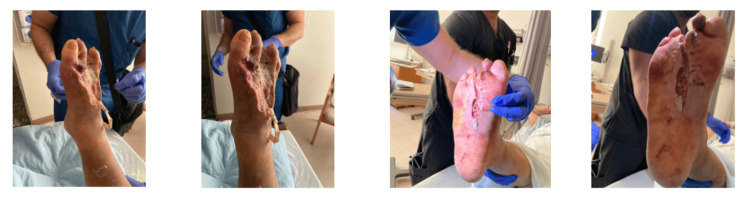
Post-second debridement images of the right foot The images demonstrate extensive plantar necrosis, third toe amputation, and serial changes in tissue viability following drainage and debridement.

Two days later, he returned to the operating room for a second debridement due to persistent drainage and concern for deeper involvement. Additional necrotic tissue was excised, and the drains were repositioned. Final wound cultures demonstrated no growth, likely reflecting prior antibiotic administration. He remained clinically stable but at continued high risk for limb loss. Inflammatory markers and renal function showed only partial improvement compared to admission, with C-reactive protein (CRP) decreasing from 287 mg/L to 247 mg/L and renal indices improving modestly (blood urea nitrogen (BUN) from 34 mg/dL to 31 mg/dL, creatinine from 1.78 mg/dL to 1.50 mg/dL). Figure 2 shows the condition of the right foot following the two surgeries.

**Figure 3:**
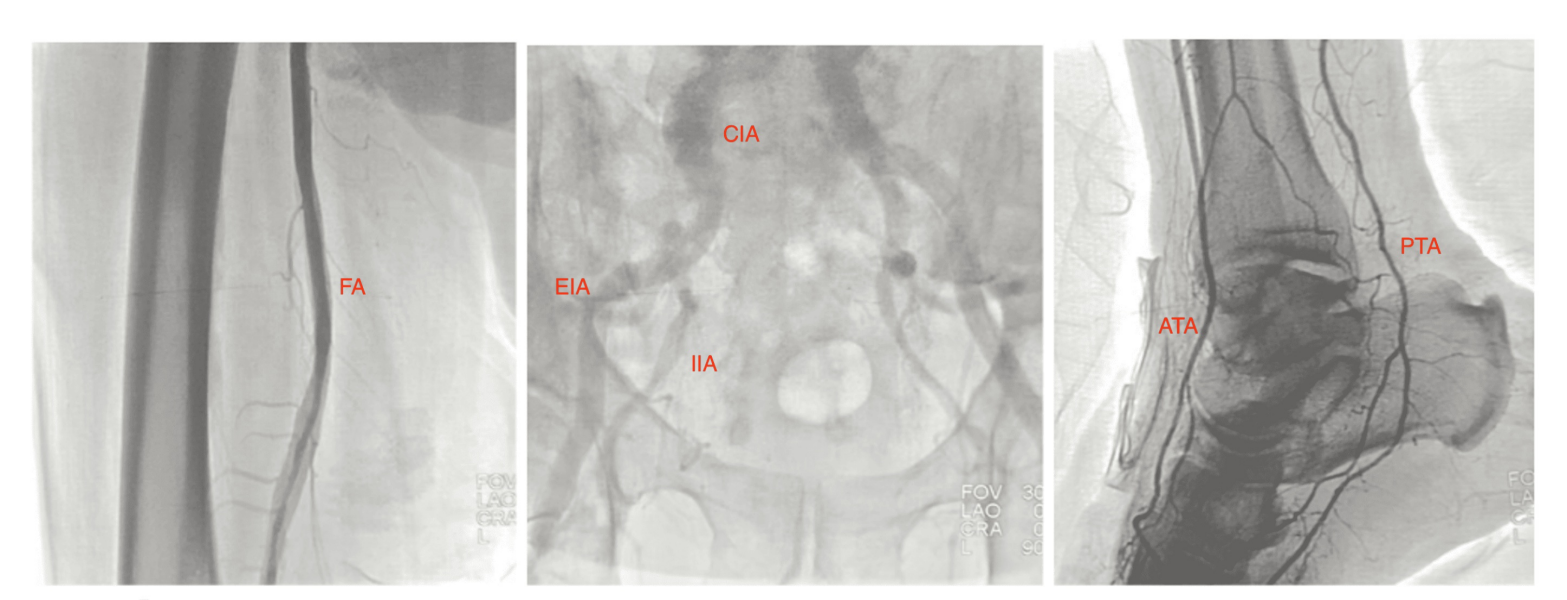
Abdominal aortogram with runoff and selective right femoral angiography demonstrating patent CIA and EIA, and FA with two-vessel runoff to the right foot through the ATA and PTA CIA: Common iliac artery; EIA: External iliac artery; FA: Femoral artery; ATA: Anterior tibial artery; PTA: Posterior tibial artery

Given his risk profile, cardiology was consulted for peripheral angiography to assess candidacy for revascularization. An abdominal aortogram with runoff and selective right femoral angiography demonstrated two-vessel runoff with excellent distal flow to the right foot and no significant disease in the iliac, femoral, or popliteal arteries (Figure 3). Based on these findings, the surgical team determined that a BKA would be technically feasible rather than requiring an AKA [6,7]. The patient elected to delay amputation and instead pursue outpatient wound care with plans to revisit the decision at a later time.

## Discussion

This case highlights several important points. When a patient presents with gas gangrene, management typically begins with IV antibiotics, followed by urgent surgical debridement. In selected cases, hyperbaric oxygen therapy may serve as an adjunct to antibiotic therapy, as the increased oxygen tension helps inhibit clostridial proliferation, reduce toxin production, and enhance host immune response, thereby contributing to infection control [1,2]. Early surgical intervention is critical for gas gangrene, as multiple prompt debridements can help slow or halt the spread of infection before it becomes uncontrollable [8].

Vascular imaging provided key information for this case. By confirming adequate arterial flow to the lower leg, the CT angiogram supported consideration of a BKA rather than an AKA. Preserving the knee offers significant functional advantages: Patients with a BKA require only about 25% more energy than normal walking compared to 60-100% more after an AKA, and they typically adapt to prosthetics more easily with better long-term mobility [4,5]. Furthermore, BKA lowers the risk of complications commonly associated with AKA, such as muscle atrophy, surgical site infections, wound dehiscence, and skin breakdown from prosthetic wear [7]. Although angiography remains the gold standard for definitive vascular mapping, it does carry risks such as contrast exposure and radiation.

Alternative diagnostic modalities also warrant discussion. Non-invasive vascular assessments such as Doppler studies, ankle-brachial index, and pulse volume recordings can evaluate limb perfusion and screen for peripheral artery disease, while transcutaneous oxygen pressure helps predict wound healing potential. When these studies suggest inadequate perfusion, interventions such as bypass grafting or stent placement may be considered. In addition, adjunctive modalities such as indocyanine green near-infrared (ICG-NIR) fluorescence imaging aid in assessing flap viability in reconstructive surgical procedures. This can become relevant in gas gangrene cases when extensive debridement leaves large soft-tissue defects [6].

Additional therapeutic options are available as wound management continues to advance. For example, after surgical debridement in gas gangrene, useful therapeutic adjuncts such as vacuum-sealed drainage and negative-pressure wound therapy (NPWT) can provide continuous removal of exudate and residual necrotic material, decrease bacterial burden, and promote tissue oxygenation and angiogenesis [2,8,9]. Several reports support the benefit of NPWT when used alongside debridement and antibiotics. In a retrospective study, Liu et al. demonstrated that vacuum sealing drainage with continuous irrigation effectively controlled infection, promoted granulation tissue formation, and contributed to higher rates of limb salvage [8]. In addition, other cases such as the one described by Qiu et al. highlight how vacuum-assisted closure can facilitate infection control and wound healing after extensive debridement [9]. Although NPWT was not used in our patient, it and other wound-care technologies have potential as adjunctive therapies following surgical debridement.

Decisions about care ultimately extend beyond available technologies. Although the patient understood the risks of delaying amputation, he preferred to pursue outpatient follow-up before committing to limb removal. In this case, even when the surgical option seemed clear from a medical perspective, given the progression of necrotizing infection, risk of systemic spread, and limited potential for functional limb salvage, the patient had personal reasons for choosing to defer surgery. Patient autonomy is a crucial part of healthcare and should always be included in the decision-making process [10].

## Conclusions

In this case, imaging guided the surgical plan by demonstrating that a BKA was a viable option. Although the patient ultimately chose to delay surgery, the situation highlights the value of balancing clinical judgment with patient autonomy in decision-making. It also reinforces the importance of vascular imaging not only in assessing tissue viability but in tailoring interventions that align with patient goals and overall well-being. Multidisciplinary coordination and informed shared decision-making remain foundational in managing limb-threatening infections.
